# Women’s experience with postpartum intrauterine contraceptive device use in India

**DOI:** 10.1186/1742-4755-11-32

**Published:** 2014-04-23

**Authors:** Somesh Kumar, Reena Sethi, Sudharsanam Balasubramaniam, Elaine Charurat, Kamlesh Lalchandani, Richard Semba, Bulbul Sood

**Affiliations:** 1 Jhpiego, New Delhi, India; 2 Jhpiego, Baltimore, USA

**Keywords:** Contraception, Complications, Family planning, Intrauterine contraceptive device, Postpartum

## Abstract

**Background:**

Postpartum intrauterine contraceptive devices (PPIUCD) are increasingly included in many national postpartum family planning (PPFP) programs, but satisfaction of women who have adopted PPIUCD and complication rates need further characterization. Our specific aims were to describe women who accepted PPIUCD, their experience and satisfaction with their choice, and complication of expulsion or infection.

**Methods:**

We studied 2,733 married women, aged 15–49 years, who received PPIUCD in sixteen health facilities, located in eight states and the national capital territory of India, at the time of IUCD insertion and six weeks later. The satisfaction of women who received IUCD during the postpartum period and problems and complications following insertion were assessed using standardized questionnaires.

**Results:**

Mean (SD) age of women accepting PPIUCD was 24 (4) years. Over half of women had parity of one, and nearly one-quarter had no formal schooling. Nearly all women (99.6%) reported that they were satisfied with IUCD at the time of insertion and 92% reported satisfaction at the six-week follow-up visit. The rate of expulsion of IUCD was 3.6% by six weeks of follow-up. There were large variations in rates of problems and complications that were largely attributable to the individual hospitals implementing the study.

**Conclusions:**

Women who receive PPIUCD show a high level of satisfaction with this choice of contraception, and the rates of expulsion were low enough such that the benefits of contraceptive protection outweigh the potential inconvenience of needing to return for care for that subset of women.

## Introduction

Family planning can avert nearly one-third of maternal deaths and 10% of child mortality when couples space their pregnancies more than two years apart [1]. Short intervals between births are linked with higher maternal and child mortality and morbidity [2]. Postpartum family planning (PPFP) is the prevention of unintended and closely spaced pregnancies through the first twelve months following childbirth [1]. Postpartum women need a range of effective contraceptive methods to be able to prevent an unplanned pregnancy, within a short interval [1,2].

Among the options available, the multi-year cost of the Copper T380A IUD makes it one of the most cost-effective contraceptive options available. The Copper T 380A intra-uterine contraceptive device (IUCD) is a highly effective, non-hormonal method that can be safely used by all women regardless of breastfeeding status during this interval. According to the World Health Organization Medical Eligibility Criteria, an IUCD can be inserted in the 48 hours postpartum, referred to here as a postpartum IUCD (PPIUCD), or after four weeks following a birth [3]. A 2010 Cochrane review concluded that PPIUCDs were a safe and effective contraceptive method. The public health benefits from PPIUCDs stemmed from the women’s increased accessibility to PPIUCDs following facility births, as PPIUCDs could be offered at health facilities after childbirth. This, in turn, decreased opportunity and other costs incurred by clients who may otherwise have to return to facilities to access contraceptive services [4].

In India, the 2005–2006 National Family Health Survey (NFHS) reported that 61% of births were spaced less than three years [5] and that 22% of married women had an unmet need for family planning. A subsequent stratified analysis suggested that 65% of women in the first year postpartum had an unmet need for family planning [6]. IUCDs are used by only two percent of current users of contraception in India [5]. Recognizing the potential impact of improved family planning programming on maternal and child health, the Government of India has committed to expanding access to family planning as part of achieving Millennium Development Goals 4 and 5, related to reduction of child and maternal mortality. In 2005, the Government of India launched the Janani Sukraksha Yojana (JSY), a conditional cash transfer scheme, to encourage the use of facilities for care at birth [7]. Since the inception of JSY, facility-based births in the public sector have increased from 700,000 in 2005 more than 11 million in 2012 [8].

With increasing numbers of women electing to give birth in health institutions, the Government of India decided to strengthen PPFP and to introduce PPIUCD services in a phased manner, with the first batch of clinician trainings, in 2009. A national training center was established at Safdarjung Hospital in New Delhi, as well as three regional training centers in Mumbai, Jabalpur, and Lucknow in 2009–2010. The provision of PPIUCDs is being rapidly scaled up in India, with facilities in at least nineteen states offering the method in 2013. Previously, concerns about the PPIUCD focused on high expulsion rates. Studies published in the nineties and early 2000 reported rates of about 9-13% [9-11]. However, lower expulsion rates have been reported more recently with improvements in insertion technique [12,13].

PPIUCDs are still emerging as a relatively new contraception choice in India. While follow-up data on complications with PPIUCD insertions were available from international sources, given the scale at which PPIUCD services are being introduced in India, it was important to generate country-based evidence on the post-insertion outcomes after the introduction of PPIUCD program. Additionally, information related to the demographic profile of women who accept PPIUCDs, the dynamics of their decision making process, their satisfaction with this method of contraception, and complications with the IUCD have not been well characterized. Therefore, we conducted a prospective, observational study of a large cohort of women who received PPIUCDs in India.

Our specific aims were to determine the demographic characteristics and decision-making among women who accepted PPIUCDs, their perception and satisfaction with PPIUCDs, and complications that occurred after insertion of PPIUCDs. The overarching aims of this study were to inform the stakeholders about the experience of the PPIUCD program in the country and provide guidance for further scaling up of the PPIUCD program in India. This publication addresses several specific areas within these overarching aims: clients’ satisfaction with PPIUCD as a family planning method; the importance of counseling services within the perspective of the overall decision making process of the client and her family for acceptance of a postpartum family planning method; and the post-insertion outcomes of the clients accepting PPIUCD services.

## Subjects and methods

### Study subjects

The study subjects consisted of 2,733 women, aged 15–49 years, who received PPIUCDs in sixteen health facilities, all hospitals, located in eight states (Assam, Bihar, Jharkhand, Madhyaradesh, Maharashtra, Rajasthan, Uttar Pradesh, Uttarakhand) and the National Capital Territory (Delhi) in India between January 2011 and December 2012. Hospitals were selected by convenience, based upon where Jhpiego programs had trained personnel in the provision of PPIUCDs with funding from the United States Agency for International Development (USAID)’s Maternal Child Health Integrated Program (MCHIP) and the Bill & Melinda Gates Foundation (BMGF). All women who received a PPIUCD and were married were invited to participate in the study. Women were enrolled in the study after standardized oral consent in the local language. Participants were interviewed prior to discharge after receiving a PPIUCD and then six weeks later.

### Data collection

Data were collected from participants using a structured questionnaire that included basic demographic information, perception of family planning, satisfaction with PPIUCD counseling and with the method itself. After six weeks, PPIUCD clients were again interviewed using a structured follow-up questionnaire that was used to collect information about the clients’ overall satisfaction with the method, problems or complications related to the method, and retention of the PPIUCD. Family Planning Counselors who received standardized training in the study protocol, collection of data, and research ethics were responsible for administering the questionnaires. The follow-up interview was generally conducted at the same health facility in which the client had received the PPIUCD. In the cases where the client did not return to the health facility for her follow-up visit, she was contacted by telephone for the follow-up interview. The Institutional Review Board of the Johns Hopkins Bloomberg School of Public Health approved the study. A letter of support was also obtained from the Ministry of Health & Family Welfare, Government of India.

### Statistical analysis

Continuous variables were reported using mean (standard deviation) and categorical variables were reported using percentages. For two group comparisons, a student *t*-test was used to compare means of continuous variables, and chi-square tests were used to compare categorical variables. Logistic regression models were used to examine the relationship between demographic and other covariates with problems or complications associated with IUCD. All analyses were conducted using Stata 10 and 12 (Stata Corporation, College Station, TX) and SAS (v.9.1.3, SAS Institute, Cary, NC) with a type 2 error of 0.05.

## Results

A total of 2,733 women were enrolled in the study after receiving a PPIUCD prior to discharge, and 1,730 women (63.3%) were interviewed at the six-week follow-up visit. Table 1 shows the demographic characteristics of study participants; women that received a PPIUCD in selected health facilities and were interviewed upon exit. Results showed that study participants were an average (SD) age of 24 (4) years, some (about one-quarter) had no formal education, and around half had one living child. Just over half (54%) of the interviewed women responded that they wanted more children. More than half of the participants were from Uttar Pradesh and Madhya Pradesh states, as expected from sample estimations.

**Table 1:** Demographic characteristics of clients who accepted Post-Partum Intrauterine Contraceptive Device (PPIUCD) and were interviewed at the time of discharge from the facilities

**Demographic characteristics**^**1**^		**Mean (SD) or number (%)**
Age, years		24 (4)
Education (%)	No schooling	582 (23)
	Primary school	494 (19)
	Secondary school	534 (21)
	Senior secondary	408 (16)
	Intermediate	269 (9)
	Graduate and above	304 (12)
Parity (%)	One	1422 (53)
	Two	890 (33)
	Three	271 (10)
	Four or more	100 (4)
Location (%)	Assam	479 (18)
	Bihar	227 (8)
	Delhi	199 (7)
	Jharkhand	92 (3)
	Madhya Pradesh	638 (24)
	Maharashtra	117 (4)
	Rajasthan	83 (3)
	Uttar Pradesh	745 (28)
	Uttarakhand	131 (5)
Fertility intentions (clients wanted more children)	Yes	1453 (58.6)
	No	1224 (45.2)
	No response/did not know	22 (0.8)
Clients who have heard of IUCD	Yes	1450 (53.5)
	No	1241 (45.8)
Clients who have used IUCD in the past (of the clients who have heard of PPIUCD) (n = 1450)	Yes	94 (6.5)
	No	1329 (91.7)

^1^Missing data (age, 22; education, 143; parity, 47).

The period during which women received counseling and other factors related to decision making are shown in Table 2. More than half of the women received PPFP counseling during antenatal care visits. About two-thirds of women had not previously used family planning methods in the past. While more than half of the women based their decision to use a PPIUCD based on discussing with multiple individuals, more than 70% of the women choosing to use a PPIUCD as a contraceptive method received PPFP counseling by a dedicated counselor at the facilities, and many stated they made the own decision to use a PPIUCD before delivery, either during antenatal care or before delivery. Nearly all women were satisfied at the time of interview about their decision to have a PPIUCD inserted. Amongst study participants, 54% had heard of IUCDs before they received PPFP counseling, and of this group, only 7% of those who had heard of IUCDs before had used an IUCD before.

**Table 2:** Counseling and decision-making about PPIUCD at baseline

**Characteristic**^**1**^		**Mean (SD) or number (%)**
Number of antenatal clinic visits		5.2 (3.1)
Period of counseling (%)	Antenatal clinic	1408 (52)
	Early labor	988 (36)
	After delivery	367 (14)
	During postpartum stay	335 (12)
Satisfied with counseling (%)	Yes	2616 (98)
	No	50 (2)
Family planning methods used in the past (%)	No methods	1827 (67)
	IUCD	389 (14)
	Injectable	6 (0.2)
	Condoms	381 (14)
	Pills	93 (3.4)
	Others	15 (0.6)
Decision-making in PPIUCD as the method of family planning (%)	Self, alone	590 (22)
	Self, after consulting with family	261 (10)
	Husband	160 (6)
	Mother	13 (0.5)
	Mother-in-law	16 (0.7)
	Sister	5 (0.2)
	Multiple options	1664 (61.5)
Counseling provider (multiple responses allowed)	ASHA	96 (96.5)
	Counselor	1942 (71.1)
	Doctor	1147 (42.0)
	Nurse	439 (16.1)
	ANM	59 (2.2)
	Aganawadi worker	45 (1.7)
Timing of decision to choose PPIUCD as family planning method (%)	Antenatal clinic	1039 (39)
	Before delivery	1021 (38)
	After delivery	610 (23)
Satisfied about decision to use PPIUCD (%)	Yes	2631 (99.6)
	No	2 (0.4)

^1^Missing data (Previously used IUCD, 1300; satisfied with counseling received, 67; satisfied with decision to use PPIUCD, 100).

The timing of PPIUCD insertion and perceptions of pain are shown in Table 3. About half of the IUCDs were post-placental insertions and nearly one-third were inserted during C-section. About three-quarters of women reported no pain at all during or after insertion. Only a small proportion of women (1-2%) reported that the insertion was painful or very painful during or after insertion. At six-week follow-up interview, the complications of expulsion or infection associated with IUCD use are shown in Table 4. The results show self-reported expulsion rate of roughly 3.8% among clients, with more than three-quarters of women reporting no complaints with their PPIUCD. Only 5.4% of women suffered from symptoms suggestive of infection after the insertion. Symptoms that were considered suggestive of infection included lower abdominal pain, fever, foul smelling/abnormal vaginal discharge, painful intercourse, and bleeding after intercourse. Other self-reported side normal effects of PPIUCD insertion included cramps and abdominal pain which 8.9% of women reported experiencing, along with 5.5% reporting minor menstrual problems. There were no cases of uterine perforation.

**Table 3:** Timing of PPIUCD insertion and perceptions of pain related to insertion in 2711 women in India

**Characteristic**		**Mean (SD) or number (%)**
Timing of PPIUCD insertion (%)	Immediately after delivery	1379 (51)
	During C-section	838 (31)
	Within 48 hours	299 (11)
	Do not know	195 (7)
Client perception of pain during insertion (%)	No pain at all	1863 (71)
	Little discomfort	631 (24)
	Somewhat painful	87 (3)
	Painful/very painful	44 (2)
Client perception of pain after insertion (%)	No pain at all	1950 (76)
	Little discomfort	487 (19)
	Somewhat painful	112 (4)
	Painful/very painful	11 (1)

**Table 4:** Findings and satisfaction with IUCD at six weeks following IUCD insertion in 1730 women in India

**Variables**^**1**^		**Number (%)**
Findings reported (%)	None	1344 (78)
	Expulsion	63 (4)
	Infection	91 (5)
	Abdominal pain	138 (8)
	Menstrual/bleeding problems	82 (5)
Medical attention sought for problems (%)	Yes	405 (77)
	No	121 (23)
Reason for removal (%)	Complications	73 (70)
	Husband is opposed	4 (4)
	Want to use other method	4 (4)
	Other	23 (22)
Woman happy with choosing PPIUCD (%)	Yes	1420 (92)
	No	125 (8)
Husband happy with choice of PPIUCD (%)	Yes	1011 (69)
	No	84 (6)
	Don’t know	367 (25)

^1^Missing data (happy with choosing PPIUCD, 185; husband happy with PPIUCD, 268.

## Discussion

This study of PPIUCD use in India showed that most women were satisfied with their choice of immediate insertion of an IUCD and that the rates of problems and complications were relatively low. Most of the women made the decision to use a PPIUCD as a method of contraception during the antenatal period or before delivery, signifying the importance of counseling in the antenatal period and before delivery. While about 54% of the women used the method for spacing their pregnancy, the remaining women accepted this method even though they did not want any more children. The large proportion of women accepting the method to limit future childbearing indicates the important place PPIUCDs hold, as a long-acting reversible method, within the basket of PPFP methods.

A majority of women (87.6%) reported the acceptance of PPIUCD as a contraceptive method due in part to the fact that it is a long acting method. Additionally, 22% of women accepting a PPIUCD cited the free-of-charge services as one reason for choosing the method. Some women, about 12.7%, also stated that they were at least partially influenced by the infrequent follow-up trips to the facility when choosing the PPIUCD, while 20.5% considered the non-hormonal nature of the method when choosing. Of importance, only half of women had heard of the IUCD before they were counseled at the facility and only 7% had ever used one. This implies that a vast majority of clients accepting PPIUCD services are first time users of IUCDs, underscoring how offering services like PPIUCDs shifts the PPFP method mix through access to long-acting reversible methods.

Nearly all women were satisfied with their choice of IUCD at the time of insertion and over 90% reported that they were happy with the IUCD at six weeks following insertion. A previous study from Orissa among interval IUD users found that about three-quarters of women were satisfied with this mode of contraception after one year [14]. The present study is somewhat suggestive that satisfaction rates are higher with PPIUCD than with interval IUD use, but the follow-up time in the two studies is not directly comparable.

In the present study, the expulsion rate was about 3.6%, which compares to the expulsion rate of 5.6% reported among 210 women in a clinic in Hubli, Karnataka state in India [15], 1.6% among 3000 women in a hospital in Paraguay [12], and 5.6% among women among 305 periurban Lusaka, Zambia [13]. Another study of 1317 women in north India reported a cumulative expulsion rate of 10.7% by six months [16]. Higher expulsion rates of around 9-16% have been reported in earlier studies [9-11,17]. One recent study from Turkey of PPIUCD among women after C-section reported an expulsion rate of nearly 18% [18]. Requests for removal of IUCD was 5.9% in the present study, compared with 7.6% reported in Hubli, India [15], 3.4% among women in Paraguay [12], and 3% among women in Zambia [13].

About 5 % of women in the present study reported infections. This rate is higher than the rate of 0.1% reported among women in Paraguay [12]. A limitation of the present study is that infection was based upon self-report and was not corroborated by medical records or microbiological confirmation. A small proportion of the study group, 120 women (3.8%), had their PPIUCD removed within the first six weeks of insertion. Women most commonly reported expected side effects of IUCDs as the reasons for the removal, including bleeding and abdominal pain. These findings suggest that there is room for strengthening PPIUCD counseling services, particularly regarding normal side effects and complications that arise from method use.

The present study is limited in that long-term expulsion rates could not be determined since follow-up was only conducted at six weeks following birth. Further studies could be conducted that involved one or two year follow-up assessments. Although the present study included a large sample of women from eight states and a territory in India, the findings cannot necessarily be generalized to all of India since the hospitals involved were a convenience sample rather than a sample representative of the country.Expansion of access to PPIUDs in India may provide an opportunity to address the high proportion of births with short intervals and improve maternal and child health outcomes. More study is needed to assess the effects of PPIUD on continuation and birth spacing in the future.

## Conclusions

Women who receive PPIUCD show a high level of satisfaction with this choice of contraception, and the rates of expulsion were low enough such that the benefits of contraceptive protection outweigh the potential inconvenience of needing to return for care for that subset of women.

## Competing interests

The authors declare that they have no competing interests.

## Authors’ contributions

SK, RS, and BS conceived the study. SB analyzed the data. SK, RS, EC, and KL drafted the manuscripts. SK, RS, and BS extensively reviewed and revised the article. All authors read and approved the final manuscript.
